# ATA homozigosity in the *IL-10 *gene promoter is a risk factor for schizophrenia in Spanish females: a case control study

**DOI:** 10.1186/1471-2350-12-81

**Published:** 2011-06-09

**Authors:** Berta Almoguera, Rosa Riveiro-Alvarez, Jorge Lopez-Castroman, Pedro Dorado, Rosario Lopez-Rodriguez, Pablo Fernandez-Navarro, Enrique Baca-García, Jose Fernandez-Piqueras, Rafael Dal-Ré, Francisco Abad-Santos, Adrián LLerena, Carmen Ayuso

**Affiliations:** 1 Genetics Department, CAIBER Unit, IIS-Fundacion Jimenez Diaz, Madrid, Spain; 2 CIBERER ISCIII, Madrid, Spain; 3 Psychiatric Department, IIS - Fundacion Jimenez Diaz, Madrid, Spain; 4 CICAB, Clinical Research Centre, CAIBER Unit, Extremadura University Hospital and Medical School, Badajoz, Spain; 5 CIBERSAM, ISCIII, Madrid, Spain; 6 Clinical Pharmacology Department, CAIBER Unit, Hospital Universitario de la Princesa, Instituto de Investigación Sanitaria Princesa (IP), Madrid, Spain; 7 CIBEREHD ISCIII, Madrid, Spain; 8 Cancer and Environmental Area, National Center of Epidemiology, ISCIII, Madrid, Spain; 9 CIBERESP, ISCIII, Madrid, Spain; 10 Psychiatrics Department, Columbia University, New York, USA; 11 Department of Cellular Biology and Immunology, CBMSO, CSIC-UAM, Madrid, Spain; 12 Department of Preventive Medicine, Public Health and Medical Immunology and Microbiology, School of Health Sciences, Rey Juan Carlos University, Madrid, Spain

## Abstract

**Background:**

Three *IL-10 *gene promoter single nucleotide polymorphisms *-1082G > A, -819C > T *and *-592C > A *and the haplotypes they define in Caucasians, *GCC, ACC, ATA*, associated with different IL-10 production rates, have been linked to schizophrenia in some populations with conflicting results. On the basis of the evidence of the sex-dependent effect of certain genes in many complex diseases, we conducted a sex-stratified case-control association study to verify the linkage of the *IL-10 *gene promoter SNPs and haplotypes with schizophrenia and the possible sex-specific genetic effect in a Spanish schizophrenic population.

**Methods:**

241 DSM-IV diagnosed Spanish schizophrenic patients and 435 ethnically matched controls were genotyped for *-1082G > A *and *-592C > A *SNPs. Chi squared tests were performed to assess for genetic association of alleles, genotypes and haplotypes with the disease.

**Results:**

The *-1082A *allele (p = 0.027), *A/A *(p = 0.008) and *ATA/ATA *(p = 0.003) genotypes were significantly associated with schizophrenia in females while neither allelic nor genotypic frequencies reached statistical significance in the male population.

**Conclusions:**

Our results highlight the hypothesis of an imbalance towards an inflammatory syndrome as the immune abnormality of schizophrenia. Anyway, a better understanding of the involvement of the immune system would imply the search of immune abnormalities in endophenotypes in whose sex and ethnicity might be differential factors. It also reinforces the need of performing complex gene studies based on multiple cytokine SNPs, including anti and pro-inflammatory, to clarify the immune system abnormalities direction in the etiology of schizophrenia.

## Background

There is consistent evidence pointing the immune system as playing an important role in both etiology and pathophysiology of schizophrenia [1,2]. A key element of this immune theory is the significant increase in the levels of some Th1 (T helper cell type 1) cytokines such as IL-1, IL-6 or TNF (tumor necrosis factor) in plasma, or even cerebrospinal fluid, found in schizophrenic patients, and abnormal levels of Th2 (T helper cell type 2) cytokines such as IL-10 [3] . It is well documented that some cytokines production is regulated at the transcriptional level, which may indicate that the immune alterations in schizophrenia could have a genetic origin [4].

This occurs with IL-10 in which three gene promoter single nucleotide polymorphisms (SNPs), *-1082G > A, -819C > T *and *-592C > A*, define three haplotypes in Caucasians, *GCC, ACC, ATA*, associated to different IL-10 production rates [5]. Both *IL-10 *SNPs and haplotypes have been reported to be in linkage disequilibrium with schizophrenia in some populations [3,6,7].

On the other hand, there is evidence of sex-specific association of certain genes with many complex diseases including schizophrenia [8] where sex differences have been described for genes such as *RELN *[9], *XBP1 *[10]*Nogo *[11] and *IL-10 *[6].

Thus, the aims of this study were to verify the linkage of the *IL-10 *gene promoter SNPs and haplotypes with schizophrenia and to explore a putative sex-specific genetic association of these polymorphisms in a Spanish schizophrenic population.

## Methods

### Subjects

241 Caucasian (64% males) unrelated Spanish schizophrenic adult patients, mean age (± Standard Deviation-SD-) 45 ± 13 years, were enrolled in the study. DSM-IV diagnosis was provided using a brief structured psychiatric interview, the Spanish version of the Mini International Neuropsychiatric Interview version 4.4 (MINI 4.4) [12].

435 unrelated adult (47% males), mean age ( ± SD) 38 ± 18 years, Caucasian, Spanish healthy volunteers, with no clinical history of chronic illness, were enrolled in the study from 3 different centers.

The study protocol was reviewed and approved by Ethics Committees of all participating centers and conducted according to the tenets of the Declaration of Helsinki. Before enrolment, all participating subjects signed an informed consent form after the study objectives and procedures were fully explained.

### DNA extraction and genotyping

Genomic DNA was extracted from 7 ml of peripheral blood samples using both automatic DNA extractors (*BioRobot EZ1, QIAGEN, Hilden, Germany; MagNA Pure System, Roche Applied Science*) and conventional salting-out methods. DNA concentration and quality of samples were assessed spectrophotometrically (*NanoDrop^® ^ND-1000 Spectrophotometer, Wilmington DE, USA*).

Genotyping of *-1082G > A *SNP was performed through a pharmacogenetic tool, *PHARMAchip*^® ^(*Progenika Biopharma, Spain*) that includes genetic variants involved in therapeutic outcome, which methodology has been described elsewhere [13].

*IL-10 -592C > A *was genotyped with a TaqMan^® ^Pre-Designed SNP genotyping assay (c__747363_10) according to the TaqMan Allelic Discrimination technology [14] using the ABI Prism 7000 *(Applied Biosystems, Foster City, CA, USA*).

Since there were two different genotyping methods, both results were further confirmed by genotyping 100 randomly selected samples for the three *IL-10 *promoter SNPs by direct DNA sequencing, using a BigDye Terminator Cycle Sequencing Kit and an ABI Prism 3130xl DNA sequencer **(***Applied Biosystems, Foster City, CA, USA*). Therefore, the *-819C > T *SNP was also assessed in these samples. Primers used for the PCR reaction were 5'GACAACACTACTAAGGCTTCTTTGG3'-forward- and 5'TGTAGGAAGCCAGTCTCTGGA3'-reverse- obtaining a PCR product of 540 bp. Cycling conditions were 95ºC for 10 minutes, followed by 35 cycles of 95ºC for 30s, 59ºC for 30s and 72ºC for 40s, with a final elongation of 10 minutes at 72ºC.

### Data analysis

*-1082G > A *variant results were translated into genotypes by Progenika. Similarly, *-592C > A *results were processed by the ABI Prism 7000 Allelic Discrimination Analysis software. Since the three *IL-10 *gene promoter SNPs are in linkage disequilibrium in Caucasians and only three haplotypes have been described (*GCC, ACC, ATA*), they were constructed according to SNPs at positions *-1082 *and -*592 *as previously described by Ortiz et al [15].

Allele, genotype and haplotype (allelic and genotypic) frequencies for both cases and controls were calculated and Hardy-Weinberg equilibrium was estimated. A sex-stratified case-control analysis was performed to find out the possible sex-specific association of *IL-10 *gene promoter SNPs to schizophrenia.

### Statistical analysis

Student T test was performed to assess for possible significant differences in age between cases and controls globally and in each sex group. Pearson's chi squared test was used to compare allele, genotype or haplotype frequencies between schizophrenics and controls in male and female populations. Test for equality of proportions was used to find possible differences in the incidence of ATA between schizophrenic and healthy females. The odds ratio (OR), with its 95% confidence interval (95% CI), was the measure of the strength of association between alleles, genotypes and haplotypes with schizophrenia in the two groups. Statistical analysis was carried using R.2.10.0 software (from *R Project for Statistical Computing *- http://www.r-project.org-).

## Results

241 patients and 435 controls were finally genotyped for SNP *-1082G > A*. The totality of DNA control samples could not be genotyped for *-592C > A*. Thus, *IL-10 -592C > A *variant and *IL-10 *gene promoter haplotypes could be determined in 241 patients and 381 controls. The results of *-1082G > A *and *-592C > A *sequencing yielded a 98% of concordance, since there were two discrepancies between methods for both variants. *-1082G > A, -592C > A *genotypes and haplotype distributions were in Hardy-Weinberg equilibrium at the 5% significant level.

Table 1 summarizes the results of the *-1082G > A *and -*592C *>*A *allelic and genotypic frequencies in the sex-stratified analysis and the mean age of each population. Mean age between cases and controls resulted significantly different globally (p = 0.000) and stratifying by sex (p = 0.002 in female case-control group and p = 0.000 in male case-control group) Neither allelic nor genotypic frequencies reached the statistical significance in the association in the male population while female schizophrenics had a statistically significant higher incidence of allele -*1082A *than the controls as well as genotype -*1082A/A*.

**Table 1:** Allelic and genotypic frequencies of -*1082G > A *and *-592C > A IL-10 *gene promoter variants

	Males						Females					
	
	Control (n = 201)		Schizophrenia (n = 153)		OR (95%CI)	P	Control (n = 234)		Schizophrenia (n = 88)		OR (95% CI)	P
	
Age (years)	37 ± 18		45 ± 13			0.000*	39 ± 18		46 ± 13			0.002*
***-1082G > A *** **Allelic frequencies**	**n**	**%**	**n**	**%**		**p**	**n**	**%**	**n**	**%**		
***G***	164	40.8%	111	36.3%	1.21 (0.88-1.66)	0.252	198	42.3%	57	32.4%	1.53 (1.04-2.25)	0.027*
***A***	238	59.2%	195	63.7%			270	57.7%	119	67.6%		
**Genotypic frequencies**												
***G/G***	38	18.9%	23	15.0%		0.502	33	14.1%	11	12.5%		0.008*
***G/A***	88	43.8%	65	42.5%			132	56.4%	35	39.8%		
***A/A***	75	37.3%	65	42.5%			69	29.5%	42	47.7%		

***-592 C > A*** **Allelic frequencies**	**n**	**%**	**n**	**%**	**OR (95%CI)**	**p**	**n**	**%**	**n**	**%**	**OR (95% CI)**	**p**
***C***	274	75.7%	227	72.8%	1.11 (0.77-1.60)	0.618	298	74.5%	123	69.1%	0.76 (0.51-1.15)	0.213
***A***	88	24.3%	79	25.3%			102	25.5%	53	29.8%		
**Genotypic frequencies**												
***C/C***	110	60.8%	84	53.8%		0.134	106	53.0%	47	52.8%		0.004*
***C/A***	54	29.8%	59	37.8%			86	43.0%	29	32.6%		
***A/A***	17	9.4%	10	6.4%			8	4.0%	12	13.5%		

-592*C > A *allelic and genotypic distribution was similar between schizophrenic and healthy males while, again, there was a statistically significant association of genotype *A/A *with schizophrenia in the females.

Table 2 shows the analysis of haplotypes stratified by sex. While allelic frequencies were similar in cases and controls in both groups, when comparing genotypic distribution, it differed significantly in schizophrenic females. Significant trends were observed for genotypes *ATA/ATA *and *GCC/ATA*. Frequencies of carrying 2, 1 or no copies of *ATA *in females, and the resultant p-value of the test for equality of proportions, are summarized in table 3 which shows a statistically significant higher incidence of 2 *ATA *in the schizophrenic females compared to the healthy ones. The ORs of association of 2 *ATA *versus 1 or no copies of *ATA *and, also, of 1 *ATA *versus 0 *ATA *(table 4) points that carrying 2 copies of *ATA *was the higher statistically significant risk factor when comparing to 1 or no copies.

**Table 2:** Allelic and genotypic frequencies of *IL-10 *gene promoter haplotypes in schizophrenic and healthy males and females

	Males					Females				
	
	Control (n = 181)		Schizophrenia (n = 153)			Control (n = 200)		Schizophrenia (n = 88)		
**Allelic frequencies**	**n**	**%**	**n**	**%**	**p**	**n**	**%**	**n**	**%**	**p**

***GCC***	146	40.3%	111	36.3%	0.557	167	41.8%	58	33.0%	0.124
***ACC***	126	34.8%	115	37.6%		134	33.5%	65	36.9%	
***ATA***	90	24.9%	80	26.1%		99	24.8%	53	30.1%	

**Genotypic frequencies**										

***GCC/GCC***	33	18.2%	23	15.0%	0.382	26	13.0%	12	13.6%	0.003*
***GCC/ACC***	48	26.5%	39	25.5%		60	30.0%	20	22.7%	
***GCC/ATA***	32	17.7%	26	17.0%		55	27.5%	14	15.9%	
***ACC/ACC***	28	15.5%	22	14.4%		22	11.0%	16	18.2%	
***ACC/ATA***	22	12.2%	32	20.9%		30	15.0%	13	14.8%	
***ATA/ATA***	18	9.9%	11	7.2%		7	3.5%	13	14.8%	

**Table 3:** *ATA *frequencies in the female subgroup

	FEMALES				
	**Control** **(n = 200)**		**Schizophrenia** **(n = 88)**		**Equality of** **proportions**

**Number of ATA alleles**	**n**	**%**	**n**	**%**	**p**

**2 *ATA***	7	3.5%	13	14.8%	0.001*
**1 *ATA***	85	42.5%	27	30.7%	0.078
**0 *ATA***	108	54.0%	48	54.5%	1

2 *ATA *means homozygosity of this allele, 1 *ATA *heterozygosity and 0 *ATA *means no copy of *ATA *in the genotype.

**Table 4:** ORs of *ATA *as risk factor for schizophrenia in females

	OR	95%CI	p-value
**2 *ATA *vs. 1 *ATA***	5.85	1.91-18.90	0.000*
**2 *ATA *vs. *0 ATA***	4.18	1.43-13.08	0.002*
**1 *ATA *vs. 0 *ATA***	0.71	0.39-1.28	0.231

## Discussion

At the genetic level, many components of the immune system have been linked to schizophrenia, through candidate-gene [3,6], pathway-based [2] and genome-wide association [1] approaches that have yielded solid evidence of the immune system involvement in schizophrenia. Specifically the recent report by Sun highlights the IL-10 signaling as a candidate pathway in schizophrenia [2].

Since first described by Bochio Chiavetto [3] the linkage between -*1082G *of the *IL-10 *gene promoter variant with schizophrenia in Italians, several studies have tried to replicate this association in various populations with conflicting results. While confirmed in a recent study [6], this trend has not been observed in other populations [16] or contradictory findings have been reported [7,17].

This study addresses the significant association of *IL-10 -1082A *allele with schizophrenia females. These results contrast to those reported in Italians [3] and Polish [6] case-control studies that found the G allele significantly increased in the schizophrenia group. Regarding the report of Bochio Chiavetto [3], the allelic frequencies of the population selected as the reference are significantly different to data published in Caucasians (TSI or CEU populations of HapMap) suggesting that the association may be due to a biased selection of the control population (most likely an incorrect matching with the cases). In the second report only paranoid schizophrenics were included in the study and significant association was only found in males [6].

Considering *IL-10 *gene promoter haplotypes, only *GCC *in Italians [3] or *GTA *in Turkish [18], both associated with high IL-10 production, have been linked to the disease and although high plasma levels of this cytokine have been reported in some studies associated to schizophrenia, it has not always been replicated [19]. Furthermore, in a recent meta-analysis in which the effect of 10 cytokines plasma levels in schizophrenia was assessed, including IL-10, only significant trends have been observed for IL-1RA, sIL-2R and IL-6, providing evidence of an inflammatory syndrome in schizophrenia [20].

This study also describes for the first time the association of *IL-10 *gene promoter *ATA/ATA *low IL-10 producing genotype linked to schizophrenia in Spanish females. Our results indicate that being homozygote for *ATA *is the main risk factor for schizophrenia in Spanish females compared to *ATA *heterozygosity or other genotypes not including *ATA*.

There is evidence of the role of IL-10 in the neurodevelopmental abnormalities found in schizophrenia [21]. IL-10 is an anti-inflammatory cytokine that regulates the inflammatory response, by inhibiting pro-inflammatory cytokine production [22], and it is constitutively expressed during fetal brain development in humans [23]. Meyer et al suggest that the genetically determined differences in IL-10 production could lead to behavioral abnormalities in the adulthood after prenatal immune challenge or innate immune imbalances. They also pointed out that not only an excess of pro-inflammatory cytokines but also an imbalance between both classes of cytokines during development might alter normal brain functions in adult life [21].

Our findings are also supported by a recent report by Sharma et al, which describe a linkage between the *ATA *haplotype and differential repression of IL-10 production, under infectious conditions, in human trophoblast [24].

On the other hand, there are several evidences of gender differences in schizophrenia regarding incidence, age of onset, disease course, therapeutic response, social and intellectual functioning and brain abnormalities [25]. Additionally, a sex-specific risk of some genes to certain diseases, including schizophrenia [8] with a number of *loci *involved, has been described [6,10,11]. There are various possible mechanisms for sex differences in gene expression, including imprinting or hormonal effects [26]. In the case of *IL-10*, or genes encoding cytokines in general, estrogen receptors are found in certain immune cells [27] responsible for IL-10 production. Direct estrogen-mediated modulation of this immune cell activity leads to changes in cytokine production [28] and can explain the gender differences of *IL-10 *as risk factor in females.

## Conclusions

Although further exploration of immune system involvement in schizophrenia is needed, our results highlight the previously described hypothesis of an imbalance towards a pro-inflammatory syndrome as the immune abnormality of schizophrenia [20]. Anyway, it should be noted that immune abnormalities are found only in a relatively small subgroup of patients [21]. A better understanding of the involvement of the immune system in schizophrenia would imply the search of immune abnormalities in what has been called endophenotypes, intermediate phenotypes between the clinical entity and susceptibility genes and so, presumably closer to relevant genes [29] in whose sex and ethnicity may be differential factors. Additionally, the results of this study reinforce the need of performing complex gene studies based on multiple cytokine SNPs, including anti- and pro-inflammatory, to clarify the immune system abnormalities direction in the etiology of schizophrenia.

## Competing interests

The authors declare that they have no competing interests.

## Authors' contributions

BA and RRA have analyzed the data, genotyped the genetic variants and performed the statistical analysis. BA has also written the manuscript. JLC and EBG have recruited and diagnosed and acquired the samples of some of the schizophrenic patients. PD and AL have recruited and genotyped some of the patients and 200 healthy volunteers and have also actively participated in the design of the protocols used. RLR and FAS have recruited 220 control volunteers, genotyped them and analyzed data. PFN and JFP have actively participated in the analysis and interpretation of genotyping data and in the statistical analysis in the schizophrenic population. RDR has contributed to the analysis and interpretation of data and has also critically reviewed the manuscript for important intellectual content. CA conceived of the study, participated in its design and coordination and helped to draft the manuscript. All authors read and approved the final manuscript. The group as a whole has contributed to the recruitment and clinical and genetic characterization of patients.

## Pre-publication history

The pre-publication history for this paper can be accessed here:

http://www.biomedcentral.com/1471-2350/12/81/prepub

## References

[B1] Stefansson H Ophoff RA Steinberg S Andreassen OA Cichon S Rujescu D Werge T Pietilainen OP Mors O Mortensen PB Sigurdsson E Gustafsson O Nyegaard M Tuulio-Henriksson A Ingason A Hansen T Suvisaari J Lonnqvist J Paunio T Borglum AD Hartmann A Fink-Jensen A Nordentoft M Hougaard D Norgaard-Pedersen B Bottcher Y Olesen J Breuer R Moller HJ Giegling I Rasmussen HB Timm S Mattheisen M Bitter I Rethelyi JM Magnusdottir BB Sigmundsson T Olason P Masson G Gulcher JR Haraldsson M Fossdal R Thorgeirsson TE Thorsteinsdottir U Ruggeri M Tosato S Franke B Strengman E Kiemeney LA Melle I Djurovic S Abramova L Kaleda V Sanjuan J de Frutos R Bramon E Vassos E Fraser G Ettinger U Picchioni M Walker N Toulopoulou T Need AC Ge D Yoon JL Shianna KV Freimer NB Cantor RM Murray R Kong A Golimbet V Carracedo A Arango C Costas J Jonsson EG Terenius L Agartz I Petursson H Nothen MM Rietschel M Matthews PM Muglia P Peltonen L St Clair D Goldstein DB Stefansson K Collier DA Common variants conferring risk of schizophrenia Nature 2009 460 744 747 19571808 10.1038/nature08186 PMC3077530

[B2] Sun J Jia P Fanous AH van den Oord E Chen X Riley BP Amdur RL Kendler KS Zhao Z Schizophrenia gene networks and pathways and their applications for novel candidate gene selection PLoS One 2010 5 e11351 10.1371/journal.pone.0011351 20613869 PMC2894047

[B3] Bocchio Chiavetto L Boin F Zanardini R Popoli M Michelato A Bignotti S Tura GB Gennarelli M Association between promoter polymorphic haplotypes of interleukin-10 gene and schizophrenia Biol Psychiatry 2002 51 480 484 10.1016/S0006-3223(01)01324-5 11922883

[B4] Boin F Zanardini R Pioli R Altamura CA Maes M Gennarelli M Association between -G308A tumor necrosis factor alpha gene polymorphism and schizophrenia Mol Psychiatry 2001 6 79 82 10.1038/sj.mp.4000815 11244489

[B5] Edwards-Smith CJ Jonsson JR Purdie DM Bansal A Shorthouse C Powell EE Interleukin-10 promoter polymorphism predicts initial response of chronic hepatitis C to interferon alfa Hepatology 1999 30 526 530 10.1002/hep.510300207 10421663

[B6] Paul-Samojedny M Kowalczyk M Suchanek R Owczarek A Fila-Danilow A Szczygiel A Kowalski J Functional polymorphism in the interleukin-6 and interleukin-10 genes in patients with paranoid schizophrenia--a case-control study J Mol Neurosci 2010 42 112 119 10.1007/s12031-010-9365-6 20393813

[B7] Yu L Yang MS Zhao J Shi YY Zhao XZ Yang JD Liu ZJ Gu NF Feng GY He L An association between polymorphisms of the interleukin-10 gene promoter and schizophrenia in the Chinese population Schizophr Res 2004 71 179 183 10.1016/j.schres.2004.01.001 15374585

[B8] Patsopoulos NA Tatsioni A Ioannidis JP Claims of sex differences: an empirical assessment in genetic associations JAMA 2007 298 880 893 10.1001/jama.298.8.880 17712072

[B9] Shifman S Johannesson M Bronstein M Chen SX Collier DA Craddock NJ Kendler KS Li T O'Donovan M O'Neill FA Owen MJ Walsh D Weinberger DR Sun C Flint J Darvasi A Genome-wide association identifies a common variant in the reelin gene that increases the risk of schizophrenia only in women PLoS Genet 2008 4 e28 10.1371/journal.pgen.0040028 18282107 PMC2242812

[B10] Chen W Duan S Zhou J Sun Y Zheng Y Gu N Feng G He L A case-control study provides evidence of association for a functional polymorphism -197C/G in XBP1 to schizophrenia and suggests a sex-dependent effect Biochem Biophys Res Commun 2004 319 866 870 10.1016/j.bbrc.2004.05.060 15184063

[B11] Tan EC Chong SA Wang H Chew-Ping Lim E Teo YY Gender-specific association of insertion/deletion polymorphisms in the nogo gene and chronic schizophrenia Brain Res Mol Brain Res 2005 139 212 216 15953657 10.1016/j.molbrainres.2005.05.010

[B12] Sheehan DV Lecrubier Y Sheehan KH Amorim P Janavs J Weiller E Hergueta T Baker R Dunbar GC The Mini-International Neuropsychiatric Interview (M.I.N.I.): the development and validation of a structured diagnostic psychiatric interview for DSM-IV and ICD-10 J Clin Psychiatry 1998 59 Suppl 20 22 33 quiz 34-57 9881538

[B13] Tejedor D Castillo S Mozas P Jimenez E Lopez M Tejedor MT Artieda M Alonso R Mata P Simon L Martinez A Pocovi M Reliable low-density DNA array based on allele-specific probes for detection of 118 mutations causing familial hypercholesterolemia Clin Chem 2005 51 1137 1144 10.1373/clinchem.2004.045203 15890894

[B14] Livak KJ Allelic discrimination using fluorogenic probes and the 5' nuclease assay Genet Anal 1999 14 143 149 10084106 10.1016/s1050-3862(98)00019-9

[B15] Ortiz J Fernandez-Arquero M Urcelay E Lopez-Mejias R Ferreira A Fontan G de la Concha EG Martinez A Interleukin-10 polymorphisms in Spanish IgA deficiency patients: a case-control and family study BMC Med Genet 2006 7 56 16803619 10.1186/1471-2350-7-56 PMC1526417

[B16] Jun TY Pae CU Kim KS Han H Serretti A Interleukin-10 gene promoter polymorphism is not associated with schizophrenia in the Korean population Psychiatry Clin Neurosci 2003 57 153 159 10.1046/j.1440-1819.2003.01095.x 12667161

[B17] He G Zhang J Li XW Chen WY Pan YX Yang FP Gu NF Feng GY Yang SL He JY Liu BX Peng YW Liu J He L Interleukin-10 -1082 promoter polymorphism is associated with schizophrenia in a Han Chinese sib-pair study Neurosci Lett 2006 394 1 4 10.1016/j.neulet.2005.06.054 16378687

[B18] Ozbey U Tug E Namli M Interleukin-10 gene promoter polymorphism in patients with schizophrenia in a region of East Turkey World J Biol Psychiatry 2009 10 461 468 10.1080/15622970802626580 19153889

[B19] Cazzullo CL Scarone S Grassi B Vismara C Trabattoni D Clerici M Cytokines production in chronic schizophrenia patients with or without paranoid behaviour Prog Neuropsychopharmacol Biol Psychiatry 1998 22 947 957 10.1016/S0278-5846(98)00059-1 9789879

[B20] Potvin S Stip E Sepehry AA Gendron A Bah R Kouassi E Inflammatory cytokine alterations in schizophrenia: a systematic quantitative review Biol Psychiatry 2008 63 801 808 10.1016/j.biopsych.2007.09.024 18005941

[B21] Meyer U Murray PJ Urwyler A Yee BK Schedlowski M Feldon J Adult behavioral and pharmacological dysfunctions following disruption of the fetal brain balance between pro-inflammatory and IL-10-mediated anti-inflammatory signaling Mol Psychiatry 2008 13 208 221 10.1038/sj.mp.4002042 17579604

[B22] Fiorentino DF Zlotnik A Vieira P Mosmann TR Howard M Moore KW O'Garra A IL-10 acts on the antigen-presenting cell to inhibit cytokine production by Th1 cells J Immunol 1991 146 3444 3451 1827484

[B23] Mousa A Kjaeldgaard A Bakhiet M Human first trimester forebrain cells express genes for inflammatory and anti-inflammatory cytokines Cytokine 1999 11 55 60 10.1006/cyto.1998.0381 10080879

[B24] Sharma S Stabila J Pietras L Singh AR McGonnigal B Ernerudh J Matthiesen L Padbury JF Haplotype-dependent differential activation of the human IL-10 gene promoter in macrophages and trophoblasts: implications for placental IL-10 deficiency and pregnancy complications Am J Reprod Immunol 2010 64 179 187 10.1111/j.1600-0897.2010.00854.x 20482524 PMC3634123

[B25] Leung A Chue P Sex differences in schizophrenia, a review of the literature Acta Psychiatr Scand Suppl 2000 401 3 38 10887978 10.1111/j.0065-1591.2000.0ap25.x

[B26] Ostrer H Invited review: sex-based differences in gene expression J Appl Physiol 2001 91 2384 2388 11641384 10.1152/jappl.2001.91.5.2384

[B27] Weusten JJ Blankenstein MA Gmelig-Meyling FH Schuurman HJ Kater L Thijssen JH Presence of oestrogen receptors in human blood mononuclear cells and thymocytes Acta Endocrinol (Copenh) 1986 112 409 414 10.1530/acta.0.1120409 3489342

[B28] Bird MD Karavitis J Kovacs EJ Sex differences and estrogen modulation of the cellular immune response after injury Cell Immunol 2008 252 57 67 10.1016/j.cellimm.2007.09.007 18294625 PMC2544631

[B29] Allen AJ Griss ME Folley BS Hawkins KA Pearlson GD Endophenotypes in schizophrenia: a selective review Schizophr Res 2009 109 24 37 10.1016/j.schres.2009.01.016 19223268 PMC2665704

